# Eighteen-Year Farming Management Moderately Shapes the Soil Microbial Community Structure but Promotes Habitat-Specific Taxa

**DOI:** 10.3389/fmicb.2018.01776

**Published:** 2018-08-02

**Authors:** Huaihai Chen, Qing Xia, Tianyou Yang, Wei Shi

**Affiliations:** ^1^Department of Crop and Soil Sciences, NC State University, Raleigh, NC, United States; ^2^College of Life Science and Technology, Henan Institute of Science and Technology, Xinxiang, China

**Keywords:** organic farming, pasture, woody plant system, 16S rRNA gene, ITS, PICRUSt

## Abstract

Soil microbes have critical influence on the productivity and sustainability of agricultural ecosystems, yet the magnitude and direction to which management practices affect the soil microbial community remain unclear. This work aimed to examine the impacts of three farming systems, conventional grain cropping (CON), organic grain cropping (ORG), and grain cropping-pasture rotation (ICL), on the soil microbial community structure and putative gene abundances of N transformations using high-throughput 16S rRNA gene and ITS sequencing approaches. Two additional systems, a forest plantation (PF) and an abandoned agricultural field subject to natural succession (SUC), were also included for better assessment of the soil microbial community in terms of variation scale and regulatory importance of management intensity vs. plant type. Farming systems significantly affected the biodiversity of soil fungi but not bacteria, with Shannon index being the lowest in ORG. Bacterial and fungal communities in three cropping systems clustered and separated from those in PF and SUC, suggesting that management practices as such played minor roles in shaping the soil microbial community compared to plant type (i.e., woody vs. herbaceous plants). However, management practices prominently regulated habitat-specific taxa. *Lecanoromycetes*, a class of *Ascomycota* accounted for ∼10% of total fungal population in ORG, but almost nil in the other four systems. ORG also enriched bacteria belonging to the phyla, *Acidobacteria, Actinobacteria, Bacteroidetes, Chloroflexi*, and *Gemmatimonadetes*. Further, PICRUSt predicted that N-cycle community compositions varied with farming systems; compared to CON, ORG and ICL were more divergent from PF and SUC. Soil pH, together with inorganic N, extractable organic C, and soil organic C:N ratio explained < 50% of the total variations in both bacterial and fungal communities. Our data indicates that while moderately affecting the overall structure of the soil microbial community, management practices, particularly fertilization and the source of N (synthetic vs. organic), were important in regulating the presence and abundance of habitat-specific taxa.

## Introduction

Conventional row crop agriculture with intensive use of synthetic chemicals has been profitable, but also generated substantially adverse impacts on soil productivity, environmental quality, and human health (Montgomery, 2007). Not only has it been a large source of non-point pollution, but also it has led to soil degradation and erosion, causing serious concerns to food sustainability. In contrast, organic farming that takes advantage of animal waste and green manure to fertilize the soil and cover crops and other techniques to control weeds, insects and disease may help mitigate the issues associated with the conventional farming. Organic farming has been shown to be more effective in reducing soil erosion and degradation than the conventional farming (Reganold et al., 1987). Mixed farming that consists of different parts of agricultural production, such as crop and livestock, also has edge in utilization of natural resource and diversification for risk management, and therefore can be a good alternative to annual cropping-based conventional farming system (Thornton and Herrero, 2015). Crop and pasture rotation in north and south America has been documented to provide numerous benefits, including enhanced crop production, organic matter buildup, and water filtration and quality; and thus this mixed farming system is promising for sustaining soil productivity (Franzluebbers et al., 2014). Compared to the conventional farming, both organic and mixed farming systems appear to be closer to natural ecosystems because they rely less on the external input of synthetic chemicals, but instead more on within-system nutrient recycling. These systems have been suggested to promote the biodiversity in the agricultural landscape (Bengtsson et al., 2005).

Generally, an ecosystem with diverse organisms will be more stable in functionality under changing environment, because many species help insure that a portion will always keep functioning. Thus, biodiversity has been considered as a buffer to reduce temporal variation of ecosystem functioning and may even enhance the overall performance of ecosystem in a long run (Yachi and Loreau, 1999). Specific features of species in an ecosystem are also important, and keynote species has long been recognized for critical roles in the overall structure and function of an ecosystem. Even so-called rare species that make up a small proportion of the entire population should not be overlooked in terms of their influences on ecosystem functioning (Hol et al., 2010; Jousset et al., 2017). Over years of investigation, a consensus has been reached on the influential impacts of farming management, such as tillage, crop rotation, fertilization, and herbicide/pesticide application on the soil microbial community structure (Bossio et al., 1998; Buckley and Schmidt, 2001; Jangid et al., 2008; Xue et al., 2013; Lienhard et al., 2014). But until recently, due to the advance of high-throughput DNA sequencing techonology, soil microbial taxa and their specific features have been linked to management practices. Hartmann et al. (2015) showed that ∼10% of bacterial and fungal operational taxonomic units (OTUs) were farming system (conventional vs. organic) specific, with more specific taxa belonging to *Actinobacteria* and *Acidobacteria* and more abundant microbial guilds for the degradation of complex compounds in organic than conventional farming. Lupatini et al. (2017) also reported that ∼ 28% of bacterial OTUs were conventional vs. organic management specific, with members of *Proteobacteria* and *Acidobacteria* being more sensitive to farming management. Unquestionably, a survey of management-sensitive microbes helps to infer the ecological preference of microbes; but more importantly, it helps to bridge the knowledge gap between microbial species composition and farming system functions.

Soil microbes play multifaceted roles in the functioning of terrestrial ecosystems, such as decomposition of organic matter, nutrient cycling, and energy flow (Nazir et al., 2009; Nielsen et al., 2011), and therefore have paramount effects on crop productivity, soil quality, and agricultural sustainability (Van Der Heijden et al., 2008). Sustainable agriculture needs to design land use and operations for cost-effective high yields as well as conservation of productive soil. Very often, management practices are combined to combat soil degradation and to sustain a satisfactory agricultural productivity. Organic fertilization and use of perennial plants in a farming system have been deemed significant in this regard. However, a detailed knowledge is lacking on what microbial species are particularly promoted or suppressed by the two practices. In this work, we conducted comprehensive and systematic comparisons on the soil microbial community structure and its putative functions among conventional cropping, organic cropping, and conventional cropping-pasture rotation systems. We also included an abandoned agricultural system subject to natural succession as a positive control. Further, a forest plantation system was included to offer intriguing comparisons with cropping systems, because forest species are the important component of the southern landscape in the United States and are popular on many farms (Mueller et al., 2002; Sydorovych et al., 2009). We hypothesized that organic farming and crop and pasture rotation were more effective in promoting biodiversity and also more robust to support habitat-specific microbes compared to the conventional farming system.

## Materials and Methods

### Farming Systems and Soil Sampling

Soil samples were taken from five systems at the Center for Environmental Farming Systems that was established in 1999 in Goldsboro, North Carolina, United States (35°22′48″ N, 78°02′36″ W). The five systems, i.e., conventional cropping system subject to best management practices (CON), integrated crop-livestock system (ICL), organic cropping system (ORG), plantation forestry (PF), and abandoned agricultural field subject to natural succession (SUC), were arranged into three blocks based on a completely randomized block design, leading to total 15 plots. Plot size varied from 0.66 to 3.64 ha, being smaller for CON, ORG, and PF and larger for ICL and SUC. Soil type was Tarboro loamy sand (mixed, thermic Typic Udipsamment) in one block and Wickham sandy loam (fine-loamy, mixed, semiactive, thermic Typic Hapludult) in the other two blocks.

Plant species and management practices differed among the five systems. Crops were rotated, including corn, soybean, wheat, grain sorghum, sunflower, and leguminous winter cover crops in CON, ORG, and the 6-year cropping phase of ICL. Tree species in PF also changed over years, including long-leaf pine (*Pinus palustris*), ash tree (*Fraxinus pennsylvanica var. Ianceolata*), and bald cypress (*Taxodium distichum*). Invaded native species in SUC included shrubs (e.g., *Solidago virgaurea minuta* and *Baccharis articulate*), small trees (e.g., *Pinus taeda*), and grasses (e.g., *Andropogon*). Fertilization was mainly made to CON, ICL, and ORG, but the application rates depended on crop requirements. In CON and ICL, for example, both corn and sorghum received ∼170 kg N ha^-1^, with 50% at planting and 50% when corn and sorghum were about 6 weeks old; wheat received ∼110 kg N ha^-1^, with 35% in January and 65% in March; and soybean received no N. Applications of P and K were based on fertilizer recommendations from the annual soil analysis of each plot. In ORG, turkey litter was used and its amount depended on crop N requirements as described above as well as N provided by cover crops. Usually, 30–40% of crop N requirements came from leguminous cover crops and rest from turkey litter. The N concentration in turkey litter was often in the range of 6.5 – 14 kg N Mg^-1^. No additional P and K were applied to the ORG, except for what contained in the turkey litter. Plant protection management was also made to CON and the cropping phase of ICL, with pre-emergent and post-emergent herbicides for all grains. The pre-emergent herbicides were S-metolachlor and atrazine for corn and sorghum, dimethylamine salt of dicamba for wheat, and S-metolachlor and metribuzin for soybean. The post-emergent herbicides were ametrin or glyphosate for corn and sorghum, but fomesafen and glyphosate for soybean. The only pesticide used occasionally for soybean in CON was the fungicide Monsoon (tebuconazole). All plots in CON, ORG and ICL occasionally received lime to adjust soil pH, based on the recommendation given by the annual soil analysis.

Thirty soil cores (2.5 cm × 10 cm) were collected randomly from each plot and pooled to form a composite soil sample on October 28, 2016 to evaluate how the soil microbial community differs among diverse ecosystems. During the time of sample collection, plant species were corn in CON and ORG, a mixture of grass species in the first-year pasture phase of ICL, bald cypress in PF, and native small trees, shrubs and grasses in SUC. We also need to mention that 3 weeks prior to soil sampling, the five systems at the CEFs were flooded for ∼ 1 week due to the Hurricane Mathew on October 8–9, causing catastrophic flooding over the Coastal plains of eastern North Carolina. Sieved soil (<2 mm) was stored at -20°C prior to DNA extraction and at 4°C prior to soil chemical analysis, respectively.

### Soil Chemical Properties

Soil total C and N were determined by dry combustion method using a Perkin-Elmer 2400 CHN analyzer (Perkin-Elmer Corporation, Norwalk, CT, United States). Soil pH was measured in water with 1:2.5 soil (g)/water (ml) ratio. Soil inorganic N (NH_4_^+^-N and NO_3_^-^-N) was analyzed using a FIA QuikChem 8000 autoanalyzer (Lachat Instruments, Loveland, CO, United States) after extraction with 0.5M K_2_SO_4_ at 1:5 soil (g)/solution (ml) ratio and filtered through Whatman #42 filter paper. Extracted soil total C in 0.5M K_2_SO_4_ was measured using TOC analyzer (TOC-5000, Shimadzu Scientific Instruments, Japan).

### DNA Extraction, Amplification and Sequencing

Soil DNA was extracted from ∼ 0.6 g soil sample using FastDNA Spin Kit for Soil (MP Bio, Solon, OH, United States) according to the manufacturer’s instructions, and then column-purified using OneStep PCR Inhibitor Removal Kit (Zymo Research, Orang, CA, United States). DNA concentrations were determined (>50 ng μL^-1^) and purity was confirmed by the ratio of absorbance at 260 and 280 nm (260/280 = 1.70–1.90) using a NanoDrop Spectrophotometer (Thermo Scientific, Wilmington, DE, United States).

PCR amplifications were made for bacterial 16S rRNA gene and fungal ITS using the primer pairs targeting V3-V4 (F319: 5′-ACTCCTACGGGAGGCAGCAG-3′ and R806: 5′-GGACTACHVGGGTWTCTAAT-3′) and ITS1-ITS2 (F_KYO2: 5′-TAGAGGAAGTAAAAGTCGTAA-3′ and R_KYO2: 5′-TTYRCTRCGTTCTTCATC-3′), respectively, with Illumina MiSeq overhang adapters (Toju et al., 2012; Klindworth et al., 2013). A 50 μL PCR reaction was comprised of 25 μL 2x KAPA HiFi HotStart ReadyMix (KAPA Biosystems, Wilmington, MA, United States), 2.5 μL of template DNA (4–20 ng μL^-1^), 2.5 μL of 10 mM of each primer, and 17.5 μL of nuclease-free water. PCR was carried out using a C1000 Touch Thermal Cycler (Bio-Rad, Hercules, CA, United States) and reaction was initiated at 95°C for 3 min, followed by 25 cycles of 30 s at 95°C, 30 s at 55°C, and 30 s at 72°C, followed by a final elongation of 5 min at 72°C. A negative control with no templates was also included in the PCR. The PCR product was cleaned up using AMPure XP beads (Beckman Coulter Genomics, Danvers, MA, United States) and eluted in 10 mM Tris pH 8.5 buffer. Then, purified DNA fragments were added at both ends with unique index (barcode) sequences using the Nextera XT Index Kit (Illumina, San Diego, CA, United States), followed by a second clean up with AMPure XP beads. An equimolar mixture of the purified 16S rRNA gene and ITS fragments were paired-end sequenced on Illumina Miseq platform (300 × 2 paired end, v3 chemistry) (Illumina, San Diego, CA, United States). The Miseq sequences were deposited on the NCBI Sequence Read Archive (SRA) database under the BioProject accession number of PRJNA477363.

Quantitative real-time PCR was also performed on each soil DNA sample with three analytical replicates (CFX96 Real-Time PCR Detection System, Bio-Rad, Hercules, CA, United States) to determine the copy numbers of 16S rRNA gene, bacterial nitrite reductase gene (nirK), bacterial ammonia monooxygenase gene (*amoA*), and nitrogenase gene (*nifH*) using the primers given in a previous study (Yuan et al., 2017), which were considered as the surrogates of the abundances of soil bacteria, bacterial denitrifiers, bacterial nitrifiers, and nitrogen fixers, respectively.

### Bioinformatics Analysis

Demultiplexed sequencing data were trimmed based on the expected amplicon size (430–470 bp for 16S rRNA gene and 180–360 bp for ITS), and filtered by the maximum error rate, 0.5% using USEARCH v9.1.13 (32 bit) (Edgar, 2010). Chimeras of trimmed and filtered sequences were identified and removed using USEARCH v6.1.544 (Beta) in QIIME (Caporaso et al., 2010). For 16S rRNA gene sequencing data, operational taxonomic units (OTUs) with 97% identity were picked up with the open reference of Greengenes database (13.8) using the method of USEARCH v6.1.544 (Beta). For ITS sequencing data, OTUs were picked up with open reference of UNITE database (12.11) using RDP (Ribosomal Database Project) method (Wang et al., 2007). It should be noted here that singletons were removed from OTUs pickup. OTUs were analyzed for alpha diversity including richness (observed OTUs) and Shannon diversity based on sequence numbers rarefied to 30,000 for 16S rRNA gene and 140,000 for ITS. Bray-Curtis distance was used to analyze beta diversity by principal coordinates analysis (PCoA).

Putative functional genes involved in N transformations were also predicted using PICRUSt (Phylogenetic Investigation of Communities by Reconstruction of Unobserved States) (Langille et al., 2013), which applies 16S rRNA gene to predict the abundance of functional genes by matching sample OTUs with reference genomes. This gene-based computational approach for assessing ecological functions has been used in various environments, including soil (Voogd et al., 2015; Zarraonaindia et al., 2015; Ashworth et al., 2017; Mushinski et al., 2018). Bacterial OTUs with genes of specific N transformations were also collected with the metagenome_contributions.py script based on Kyoto Encyclopedia of Genes and Genomes (KEGG) orthology (KO) database. The accuracy of PICRUSt prediction for each soil sample was evaluated using NSTI (i.e., weighted nearest sequenced taxon index) that measures the relatedness of OTUs in a given sample to reference genomes. The NSTI score of our samples was 0.20 ± 0.01 (standard deviation), close to the value for making reliable predictions (Langille et al., 2013).

### Statistical Analysis

Analysis of variance (ANOVA) of a completely randomized block design (SAS 9.3, SAS Institute Inc., Cary, NC, United States) was used to assess significant differences in soil chemical properties and the abundances of microbial taxonomic and functional genes determined by qPCR. Microbial alpha diversities among the five systems were compared using the Monte Carlo method with 999 permutations, and beta diversities were compared using the pairwise test of Permanova method with 999 permutations. The relative abundances of microbial taxa parsed through QIIME or PICRUSt were assessed for significance among the five systems through the Kruskal Wallis method.

Pearson’s correlation was used to evaluate if the relative abundances of functional genes predicted by PICRUSt could reliably represent the gene abundances determined by qPCR. Pearson’s correlation was also performed to examine relationships between alpha diversity metrics and soil properties. DistLM (distance-based linear model) in PRIMER (Plymouth Routines in Multivariate Ecological Research Statistical Software, v7.0.13, PRIMER-E Ltd, United Kingdom) was used to evaluate the associations of soil properties (or system attributes) with bacterial and fungal beta diversities, using forward procedure to add one soil property at a time to the model and soil property chosen at each step to produce the greatest improvement in adjusted *R*^2^ (Anderson, 2004). Heat maps were made using R-package (R version 3.3.2, a language and environment for statistical computing; R Foundation for Statistical Computing, Vienna, Austria) to show all taxonomic groups that differed significantly (*P* < 0.05) among the five systems. Further, Spearman’s Rho correlation was performed to evaluate the relations between significantly different taxa and system (or management) attributes.

## Results

### Bacterial and Fungal Communities

**FIGURE 1:**
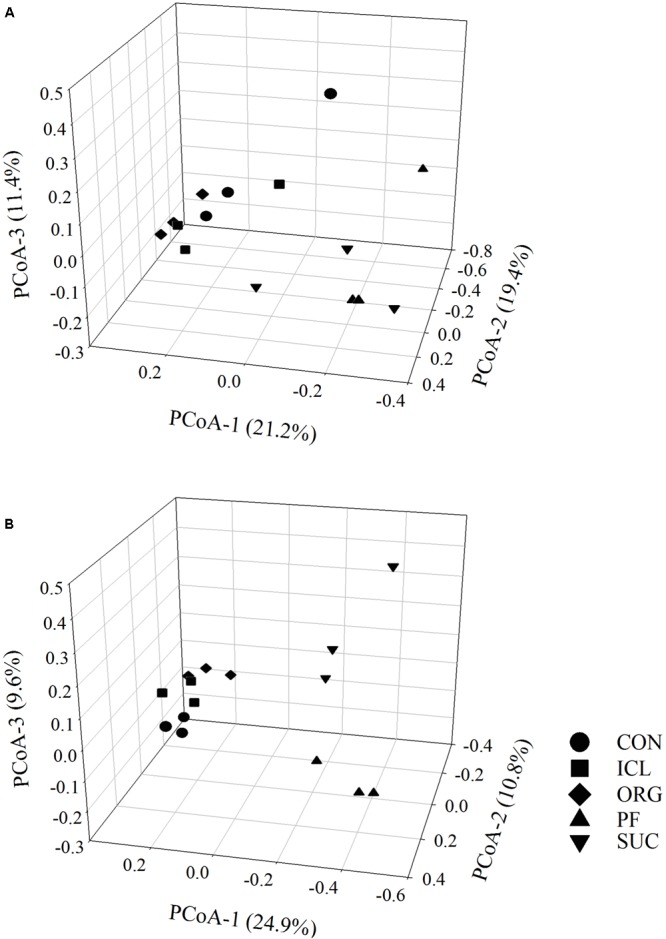
Principal coordinates analysis (PCoA) of bacterial **(A)** and fungal **(B)** communities in five systems including conventional cropping (CON), integrated crop-livestock (ICL), organic cropping (ORG), plantation forestry (PF), and natural succession (SUC). Variation explained by each principal coordinate dimension is given in percentage.

Bacterial alpha-diversity metrics (i.e., observed OTUs and Shannon diversity index) did not vary with the systems, but fungal ones differed substantially (**Table 1**, *P* < 0.05). Fungal species richness (i.e., observed OTUs) was the greatest in PF, followed by ICL, SUC, and ORG, and the lowest in CON. Fungal Shannon diversity index was also lower in ORG than the other four systems. PCoA analyses showed that bacterial and fungal community compositions and structures were system specific (**Figure 1**, *P* < 0.05). PF and SUC were well separated from CON, ICL, and ORG (*P* < 0.001); and among three cropping systems, i.e., CON, ICL, and ORG, CON also differed from ICL and ORG (*P* < 0.05), particularly for the fungal community (*P* < 0.01).

**Table 1:** Richness and diversity of bacterial and fungal communities in the five ecosystems, i.e., conventional cropping (CON), integrated crop-livestock (ICL), organic cropping (ORG), plantation forestry (PF), and abundance agricultural field subject to natural succession (SUC).

	Observed OTUs^#^		Shannon diversity	
	Bacteria	Fungi	Bacteria	Fungi
CON	8687 a	3397 c	11.8 a	7.9 a
ICL	8912 a	3990 ab	11.9 a	8.0 a
ORG	8762 a	3445 bc	11.9 a	7.1 b
PF	8582 a	4143 a	11.8 a	8.2 a
SUC	8107 a	3885 abc	11.5 a	7.8 a

*Different letters within each column indicate significant differences at *P* < 0.05. ^#^Richness and diversity metrics were estimated from a sequence depth of 30,000 for bacteria and 140,000 for fungi.*

**FIGURE 2:**
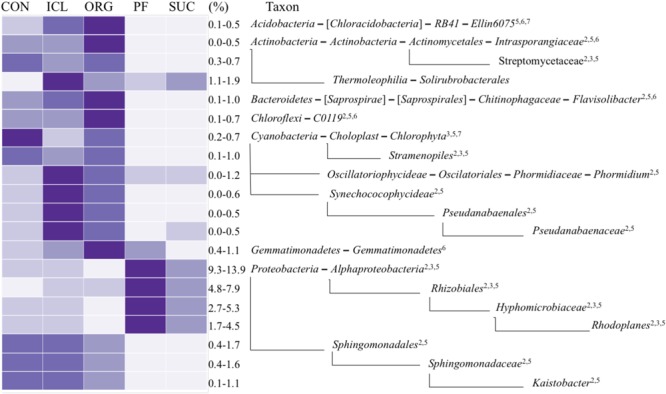
A heat map of bacterial taxa that differed significantly among the five systems (CON, ICL, ORG, PF, and SUC). The color scale is rank-based, with the darkest and lightest colors representing the highest and lowest relative abundance of each taxon among the five systems, respectively, and this range is given in percentage. Only taxa of ≥0.5% in one of the five systems are included, and taxonomic classification starts from the phylum level. The superscript numbers denote marginal (*P* ≈ 0.08) and significant (*P* < 0.05) Spearman correlations with (1) plant species diversity, (2) plant type (woody vs. herbaceous), (3) plant type (annual vs. perennial), (4) plant protection management, (5) N fertilization, (6) manure input, and (7) physical disturbance.

Of total 40 bacterial phyla detected in the five systems, a few were dominant; *Proteobacteria* accounted for 20–26% of total bacterial sequences, followed by *Bacteroidetes* 10–15%, *Acidobacteria* 8–13%, *Actinobacteria* ∼10%, *Planctomycetes* ∼9%, and *Chloroflexi* 4–9%. The relative abundances of these major phyla were similar in the five systems, except that *Acidobacteria* was marginally greater (*P* = 0.07) in PF and SUC than CON, ICL and ORG. However, sublevel taxa (i.e., class, order, family and genus) varied significantly with the systems (**Figure 2**). The class *Alphaproteobacteria* and its order *Rhizobiales* of N fixation were more abundant in PF and SUC than in CON, ICL and ORG (**Figure 2**). Specifically, a rhizobium family *Hyphomicrobiaceae* and its genus *Rhodoplanes* showed more copious in PF and SUC than in CON, ICL, and ORG. By contrast, N-fixing species of *Cyanobacteria* were generally more plenteous in CON, ICL, and ORG than in PF and SUC. Obviously, most bacterial taxa with significant differences among the five systems showed a clear dichotomy in the relative abundance between woody plant systems (i.e., PF and SUC) and herbaceous plant systems (i.e., CON, ICL, and ORG). Still, a few did not follow this trend, including the class *Gemmatimonadetes* and the order *Solirubrobacterale*. Even within the three herbaceous plant systems, ICL and ORG were more alike in the relative abundance as compared with CON. Nonetheless, there are a total of 295 bacterial taxonomic groups from phylum to genus level with >0.1% relative abundance, but only 12% showed significant differences among systems (*P <* 0.05).

**FIGURE 3:**
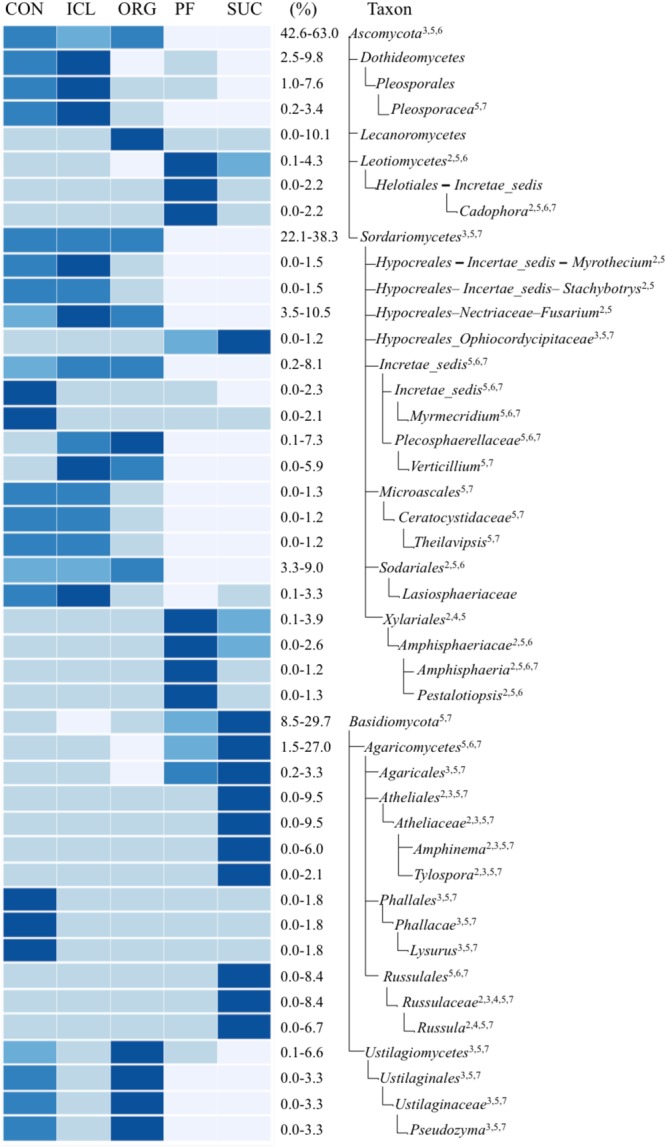
A heat map of fungal taxa that differed significantly among the farming systems (CON, ICL, ORG, PF, and SUC). The color scale is rank-based, with the darkest and lightest colors representing the highest and lowest relative abundance of each taxon among the five systems, respectively, and this range is given in percentage. Only taxa of ≥1.0% in one of the five systems are included, and taxonomic classification starts from the phylum level. The superscript numbers denote marginal (*P* ≈ 0.08) and significant (*P* < 0.05) Spearman correlations with (1) plant species diversity, (2) plant type (woody vs. herbaceous), (3) plant type (annual vs. perennial), (4) plant protection management, (5) N fertilization, (6) manure input, and (7) physical disturbance.

There were more differences in the fungal community composition among the five systems. Of total 259 fungal taxonomic groups from phylum to gene level with >0.1% relative abundance, 36% differed significantly (*P* < 0.05) and variation occurred at all the taxonomic levels (**Figure 3**). *Ascomycota* was significantly greater in three herbaceous plant systems, CON, ICL, and ORG (59–63%) than the two woody plant systems, PF and SUC (43–47%) (*P* < 0.05). By contrast, *Basidiomycota* was most abundant in SUC, accounting for 30% of total fungal abundance, followed by PF 15% and three herbaceous plant systems ∼10%. While the relative abundances of these fungal phyla mirrored the perceived dichotomy between woody plant systems and herbaceous plant systems, some sublevel taxa showed noticeable differences in the relative abundance within the three herbaceous plant systems (CON, ICL, ORG). For example, *Lecanoromycetes*, the largest class of lichenized fungi was more abundant in ORG, accounting for 10% of total fungal sequences, whereas *Pleosporales*, the largest order in the fungal class *Dothideomycetes* and with many species as saprophytes for decaying plant materials, was more abundant in ICL and CON than in ORG. *Ustilagiomycetes*, the class of true smut fungi and plant parasites, were greater in ORG and CON than ICL.

### Predicted Bacterial Gene Abundances Involved in N Cycle

Bacterial taxa involved in N transformations were sequence searched against KEGG database, using KEGG identifier, K10535 (hao, hydroxylamine dehydrogenase for nitrification), K00368 (nirK, nitrite reductase for denitrification), K00376 (nosZ, nitrous oxide reductase for denitrification), and K02588 (nifH, nitrogenase iron protein NifH for N fixation). This search resulted in a NSTI score of ∼0.2, indicating that bacterial sequences in our samples were ∼80% on average related to the sequenced genomes in the KEGG database. Although the NSTI score of our samples was close to the value for making reliable predictions (Langille et al., 2013), we further assessed the PICRUSt’s reliability using qPCR. Significant correlations (Pearson’s correlation coefficient, *r* = 0.54 – 0.76, *P* < 0.05) were found between the relative abundances of respective genes encoding enzymes for nitrification and denitrification predicted by PICRUSt and determined by qPCR.

**FIGURE 4:**
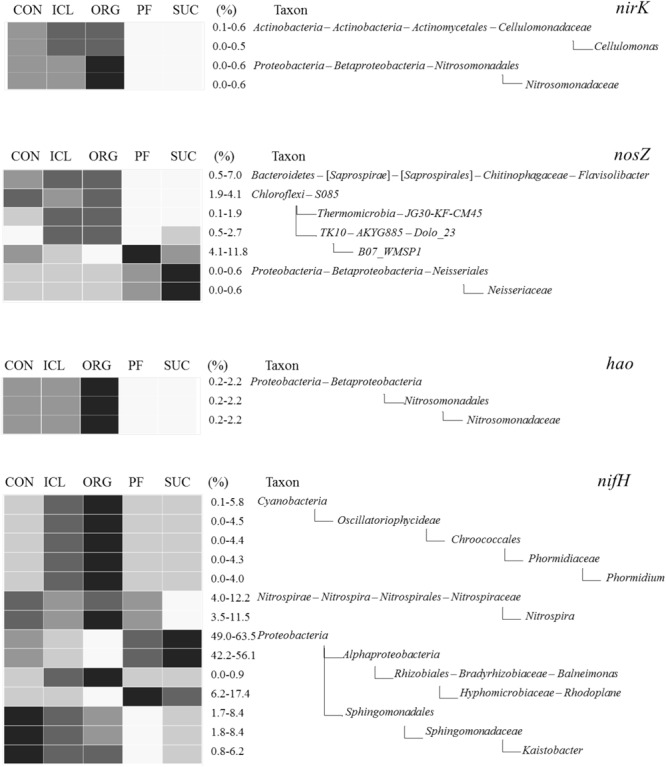
Heat maps of bacterial taxa that were predicted to be involved in denitrification (*nirK* and *nosZ*), nitrification (*hao*) and N fixation (*nifH*) and differed significantly among the five systems (CON, ICL, ORG, PF, and SUC). The color scale is rank-based, with the darkest and lightest colors representing the highest and lowest relative abundance of each taxon among the five systems respectively, and this range is given in percentage. Only taxa of ≥0.5% in one of the five systems are included, and taxonomic classification starts from the phylum level.

The four N-transformation communities differed significantly in dominant phyla (Supplementary Figure S1). The *hao* community was dominated by *Plantomycetes, Proteobacteria*, and *Nitrospirae*; *nosZ* by *Proteobacteria, Chloroflexi, Bacteroidetes*, and *Verrucomicrobia*; *nirK* by *Verrucomicrobia, Proteobacteria, Nitrospirae, Actinobacteria, Chloroflexi*, and *Bacteroidetes*; and *nifH* by *Proteobacteria, Nitrospirae, Verrucomicrobia*, and *Gemmatimonadetes*. Similar to the overall bacterial community, the composition and structure of N-transformation communities also shifted with systems (Supplementary Figure S2). Differences were found between woody plant and herbaceous plant systems as well as among the three herbaceous systems (**Figure 4** and Supplementary Figure S2). ORG was most abundant in *Nitrosomonadaceae* for nitrification and also nitrite reduction, *Flavisolibacter* and *S085* for nitrous oxide reduction, and *Phormidium* for N fixation. In contrast, *Kaistobacter* predicted for N fixation was richer in CON than the other systems.

### Relationships of the Soil Microbial Community With Soil and System Properties

Despite an 18-year history of different plant species and management practices, soil C and N were statistically insignificant among the five systems (**Table 2**). However, soil C:N ratio and extractable organic C differed significantly, being greater in SUC (*P <* 0.05). Soil pH was also different, being more acidic in the two woody plant systems compared to the three herbaceous plant systems. Inorganic N was about threefold greater in ORG than PF.

**Table 2:** Selected soil chemical properties in the five systems, i.e., CON, ICL, ORG, PF, and SUC.

	CON	ICL	ORG	PF	SUC
Soil C (g kg^-1^)	10.8 a	14.5 a	11.1 a	9.9 a	8.9 a
Soil N (g kg^-1^)	0.9 a	1.3 a	1.0 a	0.9 a	0.7 a
Soil C: N ratio	12.3 ab	11.0 b	11.2 ab	11.5 ab	13.0 a
Soil pH	6.18 a	6.20 a	6.28 a	5.60 b	5.73 b
Inorganic N (mg kg^-1^)	3.75 ab	3.48 ab	5.84 a	1.87 b	3.39 ab
Extractable organic C (mg kg^-1^)	49.0 b	45.7 b	46.7 b	53.3 ab	59.8 a

*Different letters within each row indicate significant differences at *P <* 0.05.*

Bacterial alpha diversity indices (i.e., observed OTUs and Shannon diversity index) were not correlated with soil properties. Neither were fungal alpha diversity metrics, except that fungal Shannon diversity index was negatively associated with soil inorganic N (*P* < 0.01). The DistLM analysis showed that soil pH was most significantly correlated with bacterial and fungal community structure (**Table 3**), followed by inorganic N and extractable organic C, and least for soil C:N ratio. Together, the four soil properties explained a significant portion of total variations in bacterial and fungal communities, being ∼48 and 39%, respectively (**Table 4**). Similarly, these soil properties contributed significantly to the structural variations of the N-transformation communities (data not shown). DistLM analysis was also performed to evaluate microbial responses to management cues. Here, individual management practices or system attributes were ranked from 1 to 3 among the five systems (Supplementary Table S1). The analysis confirmed that plant type (woody vs. herbaceous) and fertilization played dominant roles in affecting the relative abundance of bacterial and fungal taxa (Supplementary Table S2). Plant species diversity appeared to have no effects on microbial community diversity and composition. Plant protection management did not affect bacterial community, but significantly affected fungal community. When DistLM analysis was run against soil properties and system/management attributes together, plant type (woody vs. herbaceous) was found to be prominent in affecting both bacterial and fungal communities (Supplementary Table S3).

**Table 3:** Results of the marginal test performed by DistLM (distance-based linear model) analysis to show the associations between the soil microbial community structure and individual soil properties^#^.

Variable	SS (trace)	Pseudo-F	*P*	Prop.
**Bacterial community**				
Soil pH	898.3	3.19	0.004	0.197
Extractable C	682.4	2.29	0.023	0.150
Inorganic N	896.8	3.18	0.003	0.197
Soil C:N	617.5	2.04	0.042	0.135
**Fungal community**				
Soil pH	7410.9	2.78	0.001	0.176
Extractable C	5603.4	2.00	0.013	0.133
Inorganic N	5515.0	1.96	0.012	0.131
Soil C:N	3779.5	1.28	0.140	0.090

*^#^Prop. denotes the proportion of explained variation.*

**Table 4:** Results of the sequential test performed by DistLM (distance-based linear model) analysis to show influential soil properties in shaping the soil microbial community^#^.

Variable	Adjusted *R*^2^	SS (trace)	Pseudo-F	*P*	Prop.	Cumul.
**Bacterial community**						
Soil pH	0.14	898.3	3.19	0.002	0.197	0.197
Inorganic N	0.22	616.7	2.43	0.027	0.135	0.332
Soil C:N	0.26	397.1	1.65	0.091	0.087	0.419
Extracted C	0.27	261.3	1.10	0.369	0.057	0.477
**Fungal community**						
Soil pH	0.11	7410.9	2.78	0.000	0.176	0.176
Inorganic N	0.13	3337.0	1.28	0.109	0.079	0.255
Soil C:N	0.14	3024.3	1.17	0.237	0.072	0.327
Extracted C	0.15	2615.3	1.02	0.469	0.062	0.389

*^#^Prop. denotes the proportion of explained variation; Cumul. means the cumulative proportion of explained variation.*

Obviously, microbial responses to soil properties, system attributes and management practices varied with taxonomic groups. Of total 20 bacterial taxonomic groups that differed significantly among the five systems (**Figure 2**), 16 tended to be correlated with plant type (woody vs. herbaceous) (Spearman correlation *r* ≈ 0.89, *P* ≈ 0.08), 18 with fertilization (Spearman correlation *r* ≈ 0.87, *P* ≈ 0.08), seven with plant type (annual vs. perennial) (Spearman correlation *r* ≈ 0.95, *P* ≈ 0.02), five with manure input (Spearman correlation *r* ≈ 0.89, *P* ≈ 0.08), and two with disturbance (Spearman correlation *r* ≈ 0.89, *P* ≈ 0.08). No individuals were significantly correlated with either plant species diversity or plant protection management. Of total 44 fungal taxonomic groups that differed significantly among the five systems (**Figure 3**), 39 was correlated with fertilization (Spearman correlation *r* ≥ 0.88, *P* < 0.08), 31 with disturbance (Spearman correlation *r* ≥ 0.89, *P* < 0.08), 17 with plant type (annual vs. perennial) (Spearman correlation *r* ≥ 0.88, *P* < 0.08), 16 with plant type (woody vs. herbaceous) (Spearman correlation *r* ≥ 0.80, *P* < 0.08), 10 with manure input (Spearman correlation *r* ≈ 0.86, *P* < 0.08), and four with plant protection management (Spearman correlation *r* ≈ 0.89, *P* ≈ 0.08). Here, we need to emphasize that soil properties, system attributes and management practices could combine to influence microbial population and assemblage, thereby leading to several system-specific taxa. For example, fertilization, together with manure input promoted *Ellin6075, Flavisolibacter, Nitrosomonadaceae*, and *Plecosphaerellaceae* (**Figures 2–4** and Supplementary Figure S1).

## Discussion

Our study exclusively used an abandoned agricultural land subject to natural succession as the positive control to assess the soil microbial communities in agro-ecosystems that differed for a long term in farming practices, such as synthetic vs. organic fertilization, crop vs. crop/pasture rotation, and grain crops vs. tree plantation. Compared to SUC, farming systems did not severely affect the bacterial and fungal species richness, and yet the soil microbial community composition and structure showed system specific. Despite introducing perennial grasses into the cropping system and/or substituting synthetic N with organic N to enhance soil N cycle, bacterial and fungal community compositions in ICL and ORG still differed substantially from those in the natural ecosystem, SUC. Even more, it seemed that the two practices enlarged differences in bacterial and fungal communities between agricultural and the natural ecosystems, specifically for putative N-transformation communities. Our data support that ICL was more effective in promoting fungal species richness compared with CON, and also proves that ICL and ORG were more robust to stimulate habitat-specific taxa. As a significant example, *Lecanoromycetes*, the class of *Ascomycota* accounted for 10% of fungal population in ORG but nil in other systems.

### Ecological Drivers for Microbial Species Diversity in Farming Systems

It is generally accepted that biodiversity will decline in a conventional farming system, but will be enhanced in a farming system with diversified practices and reduced off-farm inputs, such as ICL and ORG (Bengtsson et al., 2005; Jackson et al., 2007; Kremen and Miles, 2012). The low planned-biodiversity (i.e., plant and animal species chosen by farmers) may exert negative impacts on the diversity of associated biota (e.g., birds, insects, and soil microbes) through resource and habitat selections. This, together with adverse effects of agrochemicals on non-target species may reduce the overall biodiversity of agricultural systems (Bengtsson et al., 2005; Jackson et al., 2007; Attwood et al., 2008). This framework appears to be true for living organisms of large sizes (e.g., birds and arthropods) (Bengtsson et al., 2005; Attwood et al., 2008). However, mixed results have been reported in terms of the soil microbial community in agricultural systems (Sugiyama et al., 2010; Li et al., 2012; Hartmann et al., 2015; Lupatini et al., 2017). Our results provided another evidence of the complex relationship between farming system and microbial diversity; farming systems did not affect bacterial diversity, but rather fungal diversity, with enhanced species richness in ICL and reduced Shannon diversity in ORG compared to CON. Certainly, inconsistencies in methods, metrics, and even management intensities in farming systems made it a challenge to draw a generalized conclusion over the impacts of agricultural intensification on the biodiversity of soil microbial community. However, the fact that species richness and Shannon diversity index in CON, ORG, ICL, and PF were not or slightly different from those in the natural ecosystem, SUC suggests that the ecological drivers for biodiversity are fundamentally different between belowground microorganisms and aboveground organisms of larger size.

Nutrient availability is one of the ecological factors that may regulate soil microbial diversity (Leff et al., 2015). However, its impacts have been found to be incongruent across numerous studies. For instance, fungal diversity was reported to either increase or decrease with soil N availability (Cox et al., 2010; Kerekes et al., 2013; Leff et al., 2015; Zhou et al., 2016; Zhang et al., 2017). One explanation considered that microbial diversity might be a unimodal or hump-shape function of nutrient availability; diversity could increase with nutrient availability to a tipping point and then decease thereafter (Kerekes et al., 2013). Another explanation considered the constrains of localized microbial distribution and chemical conditions on the impacts of N availability (Mueller et al., 2015). Nevertheless, our data imply that the relatively high inorganic N content in ORG led to the reduction in fungal Shannon diversity.

None of the examined soil chemical properties could explain the variation in fungal species richness among the five systems, suggesting that factors other than resource availability might be more important in dictating microbial species richness. Spatial heterogeneity at a small scale, often referred to aggregates and microsites and imparted by the number, size, and connectivity of pores, has been considered as a fundamental driver of promoting microbial species coexistence through immense resource partitioning and large reduction of competition pressure (Ettema and Wardle, 2002; Vos et al., 2013). The more abundant and diverse aggregates and microsites the soil has, the more likely microbes will survive competitive exclusions. Often, organic matter input and lack of physical disturbance are expected to favor the formation and stability of soil aggregates. Plants also contribute largely to the formation and stability of soil aggregates (Angers and Caron, 1998). Compared to CON, other systems were either less physically disturbed or had greater organic matter input. Thus, species richness was found to be the greatest in PF and ICL, followed by SUC and ORG, and lowest in CON. Given the innate character of fine-scale soil heterogeneity, however, the overall impacts of farming systems on microbial species richness and Shannon diversity were modest, with <10% of coefficient of variation.

Agreed with Hartmann and Widmer (2006), our results supported that microbial community structure was sensitive to farming practices. Both abiotic and biotic factors can strongly dictate microbial assemblages, including vegetation type and soil properties. Very often, these factors are non-independent and showed excessive correlations. In our study, ecosystems that differed in vegetation type (woody vs. herbaceous plants) also had distinct pH values, with PF and SUC being more acidic than CON, ICL, and ORG. As such, it is a challenge to disentangle their relative impacts on microbial community compositions. Data analysis, however, showed that plant type (woody vs. herbaceous plants) explained a greater portion of fungal community compositional variations (23.8%) than soil pH did (17.6%) and was the most important variable in explaining both bacterial and fungal community variations, suggesting its dominance in shaping the soil microbial community composition. The greater similarity of PF with SUC than with CON, ICL and ORG presented robust evidence that vegetation type was the key driver to assemble soil microorganisms. PF was the only system that could be considered as the monoculture, because even CON, ICL, and ORG had several crops and/or grasses to rotate over years. Although PF did not receive fertilizers and pesticides, it was subjected to extensive physical disturbance during planting and harvesting. Yet, substantial differences in plant species diversity and physical disturbance between PF and SUC did not constrain PF to recover to SUC in both α- and β-diversities of bacterial and fungal communities. Our results are in line with the inference generated from a meta-analysis that microbial community could strongly respond to vegetation types (Trivedi et al., 2016).

Plant litter biochemistry appeared to exert selection pressures on microbial taxa; *Basidiomycota*, specifically its class, *Agaricomycetes*, woody material degraders, were more abundant in SUC and PF than in the other herbaceous farming systems. *Acidobacteria* and *Alphaproteobacteria*, often perceived as oligotrophic or K-strategy organisms (Fierer et al., 2007; Kielak et al., 2016), also adapted better to the poor-resource conditions in PF and SUC, whereas copiotrophic or r-strategy organisms such as the fungal phylum *Ascomycota* (Yao et al., 2017) were less abundant in PF and SUC. Besides plant litter biochemistry, off-farm N input as manure and synthetic chemicals could also help stimulate *Ascomycota* (Nemergut et al., 2008; Leff et al., 2015), thereby leading to its dominance in CON, ICL, and ORG.

Environmental factors that shape the bacterial community likely differ from those for the fungal community (Lauber et al., 2008). By examining the two community compositional changes across a wide range of land use types (forest planation, forest, cultivated fields, and pasture land), the authors showed that soil pH and texture were responsible for variations in the bacterial community, whereas soil nutrients, including organic matter biochemistry were the main driver for the divergence of fungal community. In our study, soil pH varied moderately among the five systems, but soil nutrients associated with plant litter chemistry and off-farm input differed more largely as shown by the coefficient of variation, ∼5% for pH, ∼12% for extractable organic C and ∼38% for inorganic N. Such different scales in ecological drivers perhaps explained why in our study, fungal communities had greater distance than the bacterial communities among five systems.

### Responses of Microbial Taxa and N-transformation Traits to Management Practices

The CEFs had been managed over 18 years, with ORG having large and periodic input of turkey litter. Surprisingly, soil organic C in ORG did not differ statistically from that in CON, suggesting the rapid decomposition of organic matter in ORG. While tillage could stimulate decomposition, it was also possible that the application of turkey litter caused the stimulation of soil organic matter decomposition, through a microbial community shift to a more thermodynamically efficient one (Arcand et al., 2017). Turkey litter could help restructure the soil microbial community by altering soil C and nutrient availabilities and also serving as an inoculant of a suite of microorganisms. Compared to CON, ORG exerted positive effects on microbes belonging to the phyla, *Acidobacteria, Actinobacteria, Bacteroidetes, Chloroflexi* and *Gemmatimonadetes*, and yet these microbes only accounted for ∼6% of the total bacterial population. Hartmann et al. (2015) also found that members of these phyla were more sensitive to long-term application of farmyard manure. However, their lifestyles and ecological functions are largely unknown. For instance, the phylum *Gemmatimonadetes* is relatively abundant in soil, accounting for ∼2% of the total bacterial population, but only a few members has been isolated during the recent decade (Janssen, 2006). It seems that *Gemmatimonadetes* can better adapt to low soil moisture (DeBruyn et al., 2011), and some can even reduce nitrous oxide, an ozone depletion and global warming gas, to N_2_ under aerobic conditions (Jones et al., 2014; Park et al., 2017). Nonetheless, the specific linkage between farming management and microbial taxa helps identify a focal group of microbes to bridge the relationship among management, microbial community and soil productivity.

The most dramatic alternation in microbial community by ORG was on *Lecanoromycetes*, a fungal class of *Ascomycota*, with ∼10% of total fungal population in ORG and almost zero in the other four systems. *Lecanoromycetes* are generally known for mutualistic associations with green algae or cyanobacteria to form composite organisms, lichens. The members of this class also appear not to have the encoding genes for fungal assimilatory NO_3_^-^ reductase, imperative for soil NO_3_^-^ retention and thus preventing NO_3_^-^ loss via leaching (Gorfer et al., 2011). Did the greater abundance of *Lecanoromycetes* in ORG than the other systems suggest a shorter supply of N to microbial and plant needs in ORG? While cautions need to be taken when extrapolating microbial abundance to ecosystem processes, it seems plausible given that turkey litter input could enhance microbial activity and proliferation, thereby increasing microbial demand of N.

When we took soil samples, ICL was just rotated into the pasture after a 6-year crop phase managed as same as CON. Still, the relative abundances of several bacterial and fungal taxa in ICL differed greatly from those in CON, suggesting that on-farm animal waste recycling and use of perennial grasses exerted strong effects in shaping the bacterial and fungal community compositions. ICL had two rotational phases with management of cropping phase resembling to CON and pasture phase similar to ORG. However, microbial diversity metrics and taxon relative abundances in ICL showed noticeable differences from those in CON and ORG, perhaps due to complex interactions among farming practices. Spearman correlation analysis also indicated that plant type, fertilization, and source of N were vital farming practices in affecting taxon abundances, whereas plant protection management had litter impacts.

Abundances of functional genes involved in denitrification (*nirK* and *nosZ*), nitrification (*hao*), and N-fixation (*nifH*) accounted for ∼5% of 16S rRNA genes, regardless of methods (qPCR or PICRUSTs) used. The relative abundances of these functional genes were not different among the five systems, suggesting that diverse management practices did not affect the population sizes of bacteria involved in N transformations, although these bacteria have different lifestyles (autotrophs vs. heterotrophs, diazotrophs vs. non-diazotrophs, and aerobes vs. facultative anaerobes). This is perhaps because soil organic N ammonification was the major control of other N processes, e.g., nitrification and denitrification, when soil samples were collected. Since there were no significant differences in soil organic C and N among the five systems, ammonification was expected to be similar. However, animal waste application played more significant roles in shaping microbial community of N-transformation bacteria, specifically for those with genes encoding for nirK, nosZ, and nifH, as beta-diversities in ORG and ICL were more divergent from CON than the dissimilarities of the entire bacterial communities. Furthermore, N-transformation community compositions in ORG and ICL were more different from PF and SUC than CON with those woody systems, emphasizing the significance of N source. This raises an important question, which one, population size or species composition, matters most in terms of mediating N transformations. Further investigation should be aimed at the active profile of functional bacteria using metatranscriptomics and metaproteomics.

## Conclusion

By including a forest plantation and an abandoned agricultural field subject to natural succession as baselines and/or contrasts, this work provided experimental evidence that management practices (i.e., fertilization, source of N, plant protection, and rotation) moderately shape the soil microbial community structure. The greater similarity of PF with SUC than with CON, ICL and ORG demonstrated that compared to vegetation type (i.e., woody vs. herbaceous plants), management practices were secondary to structure the soil microbial community. However, cropping-pasture rotation was more effective in improving fungal species richness compared with the conventional cropping system. Organic cropping and cropping-pasture rotation were also robust to promote habitat-specific taxa due to long term manure application and use of perennial plant species. This work is significant, as it identifies management-associated specific taxa and thus offers an opportunity to link microbial taxa to their ecological significance.

## Author Contributions

HC and WS contributed to experimental design and data analyses. All authors contributed to the manuscript writing.

## Conflict of Interest Statement

The authors declare that the research was conducted in the absence of any commercial or financial relationships that could be construed as a potential conflict of interest.
