# Supplementary material for: Eighteen-Year Farming Management Moderately Shapes the Soil Microbial Community Structure but Promotes Habitat-Specific Taxa
Source: Front Microbiol. 2018 Aug 2;9:1776. doi: 10.3389/fmicb.2018.01776 (PMC6083213; doi:10.3389/fmicb.2018.01776)
Supplement: Supplementary file 1 [file Data_Sheet_1.docx]

Supplementary Table S1. A rank chart to show the presence/absence and the extent of management (or system) attributes for the five systems (CON, ICL, ORG, PF, and SUC). Number and color scale are ascending from absence (or less degree) to presence (or high degree).

|  | CON | ICL | ORG | PF | SUC |
| --- | --- | --- | --- | --- | --- |
| Plant species diversity | 2 | 2 | 2 | 1 | 3 |
| Plant type (woody vs. herbaceous) | 1 | 1 | 1 | 2 | 2 |
| Plant type (annual vs. perennial) | 1 | 2 | 1 | 3 | 3 |
| Plant protection management | 3 | 2 | 1 | 1 | 1 |
| Fertilization | 2 | 2 | 2 | 1 | 1 |
| Manure input | 1 | 2 | 3 | 1 | 1 |
| Physical disturbance | 3 | 3 | 3 | 2 | 1 |

Supplementary Table S2. Results of the marginal test performed by DistLM (distance-based linear model) analysis to evaluate microbial community responses to system and management cues^#^

| Variable | SS (trace) | Pseudo-F | P | Prop. |
| --- | --- | --- | --- | --- |
| Bacterial Community | | | | |
| Plant species diversity | 234.3 | 0.70 | 0.731 | 0.051 |
| Plant type (woody vs. herbaceous) | 931.0 | 3.34 | 0.003 | 0.204 |
| Plant type (annual vs. perennial) | 859.8 | 3.02 | 0.005 | 0.189 |
| Plant protection management | 451.2 | 1.43 | 0.152 | 0.099 |
| Fertilization | 931.0 | 3.34 | 0.003 | 0.204 |
| Manure input | 899.2 | 3.19 | 0.003 | 0.197 |
| Physical disturbance | 779.2 | 2.68 | 0.010 | 0.171 |
|  | | | | |
| Fungal Community | | | | |
| Plant species diversity | 3842.9 | 1.31 | 0.136 | 0.091 |
| Plant type (woody vs. herbaceous) | 10026.0 | 4.07 | 0.000 | 0.238 |
| Plant type (annual vs. perennial) | 9038.4 | 3.56 | 0.000 | 0.215 |
| Plant protection management | 5893 | 2.12 | 0.010 | 0.140 |
| Fertilization | 10026 | 4.07 | 0.000 | 0.238 |
| Manure input | 6681.0 | 2.45 | 0.004 | 0.159 |
| Physical disturbance | 8819.5 | 3.45 | 0.000 | 0.210 |

^#^Prop. denotes the proportion of explained variation

Supplementary Table S3. Results of the sequential test performed by DistLM (distance-based linear model) analysis to show influential soil properties and system/management attributes in shaping the soil microbial community ^#^

| Variable | Adjusted R^2^ | SS (trace) | Pseudo-F | P | Prop. | Cumul. |
| --- | --- | --- | --- | --- | --- | --- |
| Bacterial community |  |  |  |  |  |  |
| Plant type (woody vs. herbaceous) | 0.14 | 931.0 | 3.34 | 0.002 | 0.204 | 0.204 |
| Inorganic N | 0.23 | 625.4 | 2.50 | 0.022 | 0.137 | 0.341 |
| Soil C:N | 0.29 | 442.3 | 1.90 | 0.052 | 0.097 | 0.438 |
| Soil pH | 0.32 | 355.4 | 1.61 | 0.100 | 0.078 | 0.516 |
| Manure input | 0.35 | 291.6 | 1.37 | 0.199 | 0.064 | 0.580 |
|  |  |  |  |  |  |  |
| Fungal community |  |  |  |  |  |  |
| Plant type (woody vs. herbaceous) | 0.18 | 10026 | 4.06 | 0.000 | 0.238 | 0.238 |
| Plant protection management | 0.22 | 4043.7 | 1.73 | 0.001 | 0.096 | 0.334 |
| Plant species diversity | 0.27 | 3842.9 | 1.75 | 0.002 | 0.091 | 0.425 |
| Plan type (annual vs. perennial) | 0.30 | 3188.2 | 1.52 | 0.049 | 0.076 | 0.501 |
| Soil C:N ratio | 0.31 | 2293.1 | 1.10 | 0.366 | 0.055 | 0.556 |

^#^Prop. denotes the proportion of explained variation; Cumul. means the cumulative proportion of explained variation.

Supplementary Fig. S1. Relative abundances of major phyla predicted to be involved in nitrification (*hao*), denitrification (*nirK* and *nosZ*), and N fixation (*nifH*) in the five systems. No differences were found at the phylum level among the five systems and thus means were presented.

Supplementary Fig. S2. Principal coordinates analysis (PCoA) of *nirK* community (A), *nosZ* community (B), *hao* community (C) and *nifH* community (D) in the five systems, conventional cropping (CON), integrated crop-livestock (ICL), organic cropping (ORG), plantation forestry (PF), and natural succession (SUC). Variation explained by each principal coordinate dimension is given in percentage.


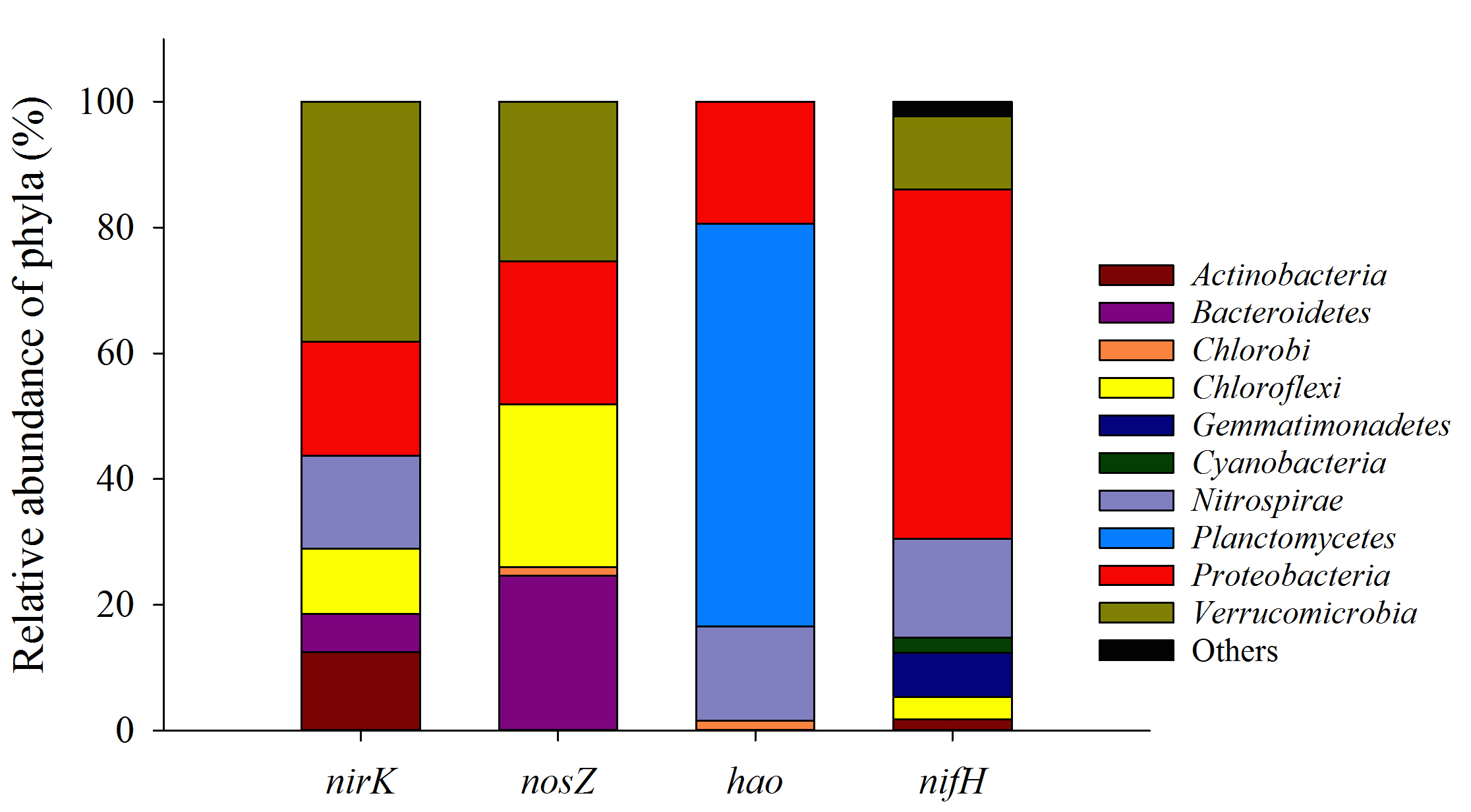


Supplementary Fig. 1


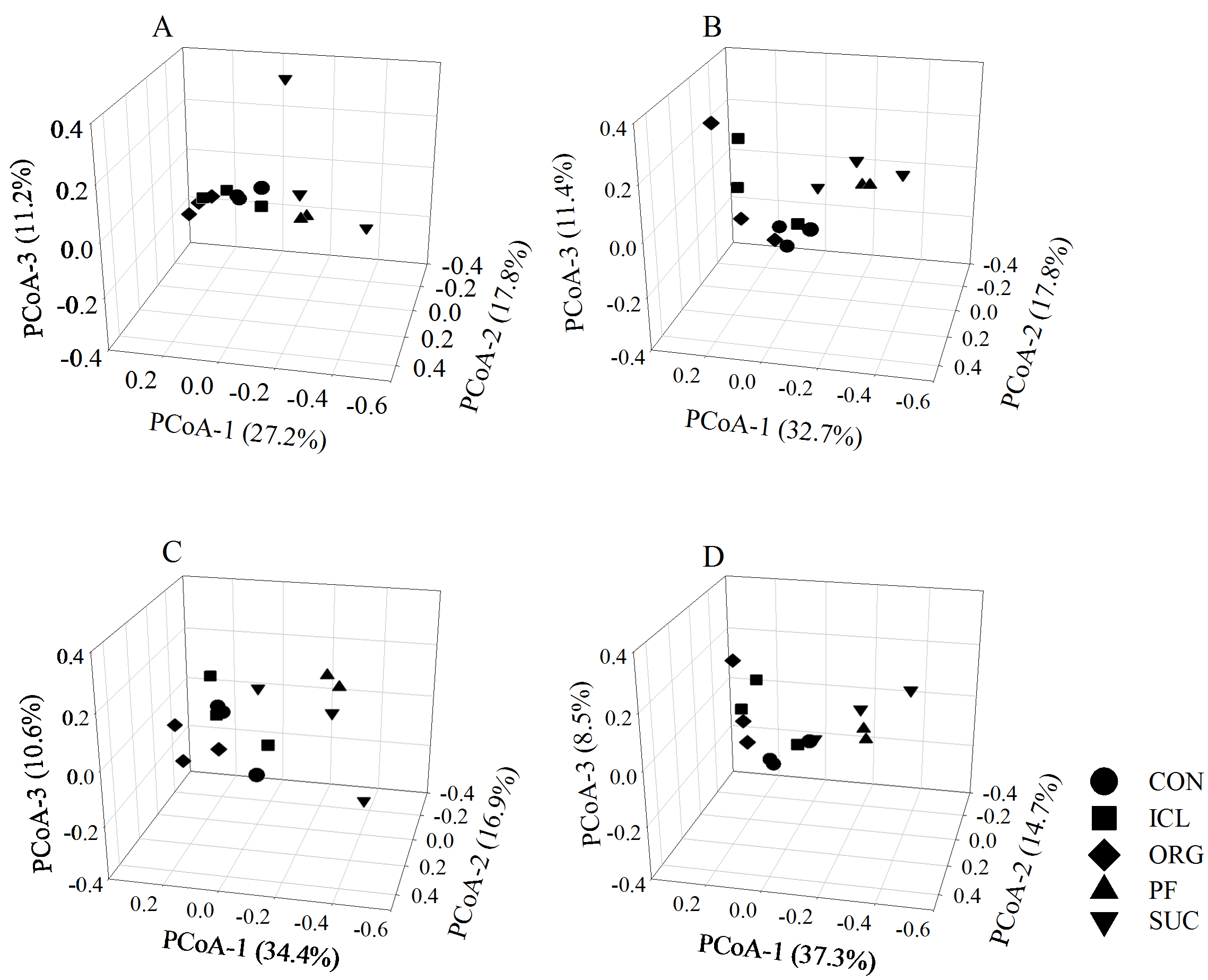


Supplementary Fig. 2
